# Potential Psychoactive Effects of Microalgal Bioactive Compounds for the Case of Sleep and Mood Regulation: Opportunities and Challenges †

**DOI:** 10.3390/md20080493

**Published:** 2022-07-29

**Authors:** Bozena McCarthy, Graham O’Neill, Nissreen Abu-Ghannam

**Affiliations:** 1 Environmental Sustainability & Health Institute (ESHI), Technological University Dublin, Grangegorman, D07 H6K8 Dublin 7, Ireland; c17731295@mytudublin.ie (B.M.); graham.oneill@tudublin.ie (G.O.); 2 School of Food Science and Environmental Health, College of Sciences and Health, Technological University Dublin, Grangegorman, D07 H6K8 Dublin 7, Ireland

**Keywords:** microalgae, bioactive compounds, polyunsaturated fatty acids, pigments, sleep, mood regulation, stress

## Abstract

Sleep deficiency is now considered an emerging global epidemic associated with many serious health problems, and a major cause of financial and social burdens. Sleep and mental health are closely connected, further exacerbating the negative impact of sleep deficiency on overall health and well-being. A major drawback of conventional treatments is the wide range of undesirable side-effects typically associated with benzodiazepines and antidepressants, which can be more debilitating than the initial disorder. It is therefore valuable to explore the efficiency of other remedies for complementarity and synergism with existing conventional treatments, leading to possible reduction in undesirable side-effects. This review explores the relevance of microalgae bioactives as a sustainable source of valuable phytochemicals that can contribute positively to mood and sleep disorders. Microalgae species producing these compounds are also catalogued, thus creating a useful reference of the state of the art for further exploration of this proposed approach. While we highlight possibilities awaiting investigation, we also identify the associated issues, including minimum dose for therapeutic effect, bioavailability, possible interactions with conventional treatments and the ability to cross the blood brain barrier. We conclude that physical and biological functionalization of microalgae bioactives can have potential in overcoming some of these challenges.

## 1. Introduction

It is estimated that between 10–30% of the world’s population suffers from insomnia [1]. Sleep deficiency is now considered an emerging global epidemic and is associated with many serious health problems, and is the cause of financial and social burden [2]. Recently, COVID-19 pandemic-related stress and anxiety have further exacerbated sleep disorders and mental health issues globally [3,4]. Sleep deficiencies are also associated with living in modern society which often entails excessive internet use and work-related stress and anxiety, resulting in lack of ‘wind down’ before bedtime and restful sleep [5,6]. Sleep difficulties can prevent falling asleep, affect sleep cycle, and cause frequent night waking. Disturbance of sleep contributes to tiredness, trouble with focus and poor concentration, low mood, frequent worrying thoughts and memory impairment [7,8]. 

Globally, approximately 17% of the adult population suffers from some form of mental health issue [9]. Sleep and mental health are closely connected. Prolonged low mood often gives rise to insomnia, lethargy, loss of appetite, feelings of stress and anxiety, which eventually can lead to depression. Lack of sleep is linked to major depressive disorders, with the risk of developing such disorders nine times higher in people with sleep deficiency [10,11,12].

Microalgae are photosynthetic organisms found to thrive in fresh and marine waters in a wide range of conditions and harsh environments, and are adaptable to temperature, pH and salinity fluctuations [13]. Microalgae are able to produce polysaccharides, proteins, lipids including polyunsaturated fatty acids (PUFAs), sterols and pigments making them attractive for nutraceutical, pharmaceutical and functional food applications. Microalgal bioactives are long known for their antioxidative, antiviral, antibacterial, anti-inflammatory, immunomodulatory and cancer and cardiovascular disease preventing properties [14,15]. 

The potential contribution of microalgae in sleep management and mental health is currently underexplored. Here, we examine the potential of microalgae bioactive compounds in management of sleep pattern, mood regulation and mental health.

## 2. Health Impact of Current Treatments for Sleep and Depressed Mood

About one third of people who suffer from insomnia take sleeping medications [16]. Benzodiazepines prescribed for insomnia and anxiety can cause loss of memory, headaches and more serious adverse effects which can increase in severity when administered along with other pharmacological interventions. For example, when taken with opioids, benzodiazepines can lead to respiratory depression, hypoxia, multiorgan damage and loss of muscle function, and can rapidly lead to dependency and increased risk of lethal overdose if abused [17,18]. Patients can develop dependency in just 3–4 weeks and tolerance leads to dose abuse, and finally to worsening insomnia, anxiety and depressed mood [19]. A study from 2019 shows that 30.6 million people in the United States take benzodiazepines, of whom 17.2% misuse the drug [20]. Medical intervention does not prevent development of depression caused by sleep insufficiency [21].

Treatments for persistent low mood and anxiety usually focus on the increase of serotonin and norepinephrine in the brain. However, antidepressants can worsen insomnia, anxiety, weight gain, aggression, sedation and drowsiness and the risk of gastrointestinal bleeding when taken with other drugs. Notably, antidepressants are associated with suicidal intention, attempt and incidence in adolescents [22,23,24,25,26,27,28].

Pharmacological solutions for treatment of insomnia and depression are no doubt beneficial; however, serious side effects and the potential to cause harm overshadow their effectiveness.

## 3. Therapeutic Potential of Microalgae in Sleep and Mood Regulation

Given the wide range of undesirable side effects associated with benzodiazepines and antidepressants, it is important to explore other available remedies. Microalgae and cyanobacteria are rich in secondary metabolites with broad health benefits. The adverse effects of therapeutics for mental health and insomnia appear to be dose dependent. The use of microalgal extracts and therapeutics in combination has seen little exploration to date. Nonetheless, microalgae can offer a promising novel approach to alleviate chronic stress, insomnia and depressed mood. There is an opportunity for future research to explore the potential of microalgae applied in conjunction with pharmacological therapies and therefore indirectly mitigate adverse side effects. The following section will explore the bioactive compounds available from microalgae in terms of their contribution towards sleep and mood regulation. A summary of microalgae species and their derivatives for sleep, mood, stress and anxiety is presented in Table 1.

### 3.1. Whole Microalgal Biomass

The use of microalgae as a potent source of bioactive compounds for health benefits is well documented. However, less data exist on microalgae biomass being beneficial for sleep, mood and mental health. Nonetheless, *Arthrospira platensis* (originally known as *Spirulina*) and *Chlorella* have been shown effective in neuroprotection, as they can protect against oxidative damage and inflammation [29,30]. Moreover, *A. platensis* was found to slightly decrease mental fatigue and improve cognitive function in clinical trials (3 g/day for seven days) [29]. Interestingly, *Chlamydomonas reinhardtii* cultures were found to generate a significant oxygen supply in the Central Nervous System (CNS) in situ. *C. reinhardtii* may be beneficial in cases of cerebral hypoxia associated with brain injury and stroke; however, in vivo confirmation is required [31].

The fresh water single cell microalgae *E. gracilis* is a source of vitamins, proteins and immunomodulatory polysaccharides such as glucans. Moreover, microalgal glucans exhibit antibacterial and cholesterol-lowering action and are effective in colorectal cancer treatment [59]. Recently, Nakashima et al. [32] studied the effects of *E. gracilis* biomass on mental health and sleep in stressed and overworked individuals. 

Prolonged stress and tension can lead to imbalance between the immune, endocrine and autonomic nervous systems. Furthermore, chronic stress is associated with depression and inflammation, and is an indicator for insomnia [11,60]. A 12-week clinical trial revealed that a daily dose of 500–1000 mg of *Euglena* had calming effects on the sympathetic nervous system and therefore promoted a more relaxed body state [32]. Similarly, the same dose of *Euglena* promoted mental wellbeing. Interestingly, the lower range (500 mg) of this dosage resulted in better response to social interaction and ability to wind down after working hours. Subjects scored higher in concentration tests and were able to focus on the task at hand better than before supplementation. Moreover, the authors suggest that *E. gracilis* acted on the autonomic nervous system, induced shorter sleep latency and improved sleep quality in a dose-dependent fashion. It should be noted that some adverse reactions, including fatigue, gastrointestinal discomfort, headache and muscle spasticity were observed in a small number of individuals. 

*E. gracilis* has the capacity to relieve anxiety, sleep difficulties and low mood [32]. However, mechanisms of action on the nervous system remain elusive. Nonetheless, *E. gracilis* is effective in mental health management and is undoubtedly worth further research. 

### 3.2. β-Phenylethylamine (PEA) 

PEA is a neurotransmitter found in cocoa beans, cheese and wine, and it can readily cross the blood–brain–barrier (BBB). Neurodevelopmental issues such as attention deficit hyperactivity disorder (ADHD) and depression are associated with PEA deficit. PEA is non-addictive, and it promotes mood balance by stimulation of serotonin, dopamine and norepinephrine production [61,62,63]. 

Marine brown and red macroalgae, as well as the green microalgae *Scenedesmus acutus,* are found to contain a wide range of PEA concentrations [64]. Cyanobacteria *Aphanizomenon flos-aquae* is rich in many secondary metabolites, proteins, phycocyanins, fatty acids, pigments and, importantly, is a PEA producer. In 2007, a dietary supplement, Klamin^®^, containing *Aphanizomenon flos-aquae* extract, was patented and is capable of providing up to 1400 µg/g of PEA [65]. Klamin^®^ shows potent antioxidant and anti-inflammatory action and is capable of promoting neuron regeneration [33]. In addition, Klamin^®^ elevated mood and lowered anxiety in menopausal women when administered at 1600 mg/day for eight weeks [66]. From a composition point of view, phycocyanins present in cyanobacteria enhance the stability and the bioavailability of PEA through the inhibition of monoamine oxidase B enzyme (MAOB), thus giving more opportunities for microalgae as producers and protectors of PEA [67].

Sabelli [68] observed that 60% of patients experienced rapid mood improvement, higher energy and concentration upon daily administration of PEA (10–60 mg) coupled with a pharmacological prescription (10 mg) for two weeks. Additionally, PEA can have a positive effect on sleep regulation and cognitive improvement, including alertness without the burden of stimulative side effects such as euphoria and tension often evoked by pharmaceutical drugs. Notably, PEA may also deliver relief in depressed patients whose symptoms did not improve using pharmacological treatment. However, the conclusion of PEA therapy most often causes mood deterioration shortly afterwards, which limits the its potential in mood regulation [68]. This highlights some of the challenges associated with nutraceutical applications; however, it also creates possibilities for alternative means of administration to harness the positive side effects.

The performance of microalgal or other sources of PEA in mental health is little explored. However, several precursors take part in the PEA synthesis and may contribute to accumulation of PEA in the brain. Essential amino acid D-phenylalanine and L-phenylalanine isomers are precursors of PEA, with the evidence of the former being more effective in depression prevention [68]. There are few studies on the relationship between oral intake of phenylalanine and PEA accumulation in the body. Dietary phenylalanine does not increase PEA plasma concentration and is considered inadequate to elicit a significant impact on its own [69]. However, when phenylalanine is administered (1–6 g/day) in combination with an antidepressant (5–10 g/day), its effect on mood increases, and it can be used in the treatment of mild depression and to prevent recurrent depression. Moreover, phenylalanine does not cause adverse effects such as irregular heartbeat as is observed with some antidepressants. This is beneficial in the elderly who have higher occurrence of death caused by slow heartbeat. Furthermore, a daily dose (1–10 g) of phenylalanine combined with 200 g of vitamin B6 prevents recurrence of depression in patients suffering from bipolar disorder [68].

Microalgae are known for generating a considerable amount of protein. Species such as *Dunaliela* sp., *S. obliquus*, *Chlorella* sp. and *Athrospira* sp. can provide more protein (40–78% dry weight) than farm animals and terrestrial crops (17.2–36% per dried matter) [70,71,72]. Phenylalanine is found in a range of foods, with higher concentrations in animal products (50 mg/g of protein) and slightly lower amounts in fruits and vegetables (40 mg/g of protein) [73]. Meanwhile, some microalgal species are capable of generating higher phenylalanine yield when compared with animals and plants, making these microorganisms more attractive for the mining of anti-depressive molecules. For example, high distribution of phenylalanine was observed in *Dunaliella* sp., for which 59.9% of its total protein content is phenylalanine, followed by *Scenedesmus* sp. (55.72%), *Nannochloropsis* sp. (55.26%) and *A. platensis* (33.3%) [74,75]. Microalgae are considered cell factories capable of generating a large range of valuable molecules. Manipulation of growth parameters, such as provision of ample nitrogen supply, can further enhance protein yield, including that of phenylalanine [76].

Bioactive compounds produced by microalgae have been shown to influence mood and sleep. Phenylalanine, found to be abundant in several microalgae, has a mild impact on depression which can be increased when coupled with antidepressants or vitamin B6. When used as a complementary therapy, PEA shows anti-depressive action and improvement in concentration and cognitive function. Microalgae produce PEA, and can therefore be exploited for production of anti-depressive molecules. 

### 3.3. Apigenin

The flavonoid apigenin is found in several terrestrial plants such as parsley, celery, oregano and chamomile flower [77]. Apigenin can penetrate the BBB and has moderate affinity for the GABA benzodiazepine binding site, thus evoking comparable sedative properties to therapeutics utilized for relaxation and sleep [78,79]. Flavonoids including apigenin are beneficial for alleviating depression and anxiety, can promote a healthy sleep cycle, improve memory and spatial learning, mitigate amnesia and are able to produce calming effects on neurons. A daily dose of apigenin ranging between 20 to 40 mg/kg showed anti-depressive effects when combined with 20 mg/kg of antidepressant in vivo [80]. 

Norepinephrine deficit is associated with sleep and memory issues, depression and anxiety [81]. Increased concentration of this neurotransmitter and inhibition of MAOB (prevention of PEA breakdown) were recorded when 12.5–25 mg/kg of apigenin was administered for seven days in vivo. A daily dose of 50 mg/kg for seven days reduced depression symptoms in rats; this was attributed to the anti-inflammatory action of apigenin [80]. Chronic stress reduction and increase in sleep length due to the sedative effects of apigenin were observed in vivo [80,82]. However, apigenin did not provide significant relief in chronic insomnia, sleep quality, and sleep latency in clinical trials [80].

Microalgae are capable of producing a broad range of valuable phytochemicals which are potent antioxidants and anti-inflammatory agents [83]. Microalgal flavonoids are more complex in their structure when compared with terrestrial plant extracts and therefore demonstrate unique properties [35]. Several antioxidative flavonoids such as apigenin have shown a neuroprotective capacity and can promote cell longevity. Several species of microalgae were found to produce apigenin, such as *Leptolyngbya* sp. (0.4 ± 0.02 mg/g), *A. platensis* (6.0 ± 0.5 ng/g), *D. lutheri* (13.6 ± 1.1 ng/g), *P. tricornutum* (7.3 ± 0.6 ng/g), *P. purpureum* (9.3 ± 0.7 ng/g), *T. suecica* (9.8 ± 0.8 ng/g), *C. vulgaris* (9.9 ± 0.8 ng/g), and *H. lacustris* (9.2 ± 0.7 ng/g) [34,35]. 

Bellahcen et al. [42] found that *A. platensis* can generate up to 21.7 mg/100 g of apigenin in ethanolic extract. This shows that *Arthrospira* has the potential to be a source of apigenin, thus making microalgae a desirable source of bioactives for low mood and stress relief. 

Microalgae generate secondary metabolites as a response to environmental stressors such as nutrient starvation and changes in light, pH and temperature. Microalgae show increased production of antioxidants in response to nitrogen restriction, salinity and light intensity fluctuations [84]. Environmental manipulation shows promise in maximising microalgal bioflavonoid output, making microalgae an advantageous bio-factory source of valuable biomolecules.

The bioflavonoid naringenin is found in citrus fruits such as grapefruits, oranges and lemons and is known to be an apigenin precursor. The prokaryotic bacterium *Streptomyces albus* has shown enhanced apigenin synthesis when fed with raw material rich in naringenin [85]. Cyanobacteria share many genetic traits with prokaryotic bacteria making these microorganisms related in DNA structure and gene distribution. Recent advancements in bioengineering techniques have made possible the utilisation of microorganisms in the production of many valuable compounds [86]. It may be possible to apply such techniques to manipulate cyanobacteria to elicit the same response to naringenin. Grapefruit peels are known to contain up to 4.1% (*w*/*w*) of naringenin [87]. Food wastage is an emerging global concern; it is therefore worth exploring if citrus peels may be utilised for the growth of microalgae while addressing the importance of circular bioeconomy. Flavonoids also have a positive effect on microalgal growth including cyanobacteria *Nostoc muscorum*, making microalgae especially attractive for apigenin production [88]. 

The evidence above suggests that microalgae are a source of molecules such as apigenin with the potential to provide benefit in stress, anxiety and mood management. Moreover, it will be worth exploring the role of precursors on apigenin productivity in microalgae. There is promise in exploring neurological responses to naringenin exposure.

### 3.4. Ferulic Acid

Polyphenols are a large class of compounds with strong radical scavenging, anti-inflammatory and many additional health promoting effects [89]. Fruits and vegetables such as elderberry, chokeberry, blackcurrant, artichoke and black bean have been long established as rich sources of polyphenols [90]. Ferulic acid is a potent anti-inflammatory phytochemical and widely distributed among plant seeds and leaves, as well as in grains and beans [91,92].

As previously mentioned, sleep deprivation may lead to low mood, poor memory and overall deterioration of mental and cognitive health. Polyphenols are involved in many signalling pathways including inflammatory control and mediation of correct functioning of cognitive function and memory [37,40]. Several studies describe the role of ferulic acid in mood regulation, stress and inflammation. Daily intragastric administration of 12.5, 25 and 50 mg/kg of ferulic acid for four weeks reduced depression symptoms in stressed rats [40]. Meanwhile, oral delivery of 20, 40 and 80 mg/kg/day for four weeks reduced inflammation associated with chronic stress and showed anti-depressive effect comparable with pharmacological treatment [93]. There is also evidence that ferulic acid can suppress genes involved in neuroinflammation response and hence can also be beneficial in the treatment of Alzheimer’s disease [40]. Interestingly, Xu et al. [94] showed that ferulic acid has anti-depressive effects when delivered in a single dose. Neurotransmitter activity was measured in vivo 30 min after oral administration of 40–80 mg/kg dosage. Rapid increase of dopamine, serotonin and norepinephrine concentration was also observed—these neurotransmitters are known to be responsible for feelings of happiness, focus, reward and positive emotions [36]. In comparison, since therapeutics would typically require several weeks to produce mood improvement, a direct analogy between ferulic acid and typical pharmaceutical drugs cannot be made. Nonetheless, ferulic acid has some capacity to exert an anti-depressive effect in vivo, and it shows potential worth pursuing. 

Fruits such as red grapes are known to be one of the richest sources of phenolic compounds and can produce polyphenol concentrations ranging from 0.52 to 4.93 mg/mL, depending on the cultivar [95]. However, the evidence suggests that microalgae are also abundant in valuable compounds and can generate polyphenol concentrations comparable to those of plants. Haoujar et al. [38] found that *Nannochloropsis gaditata*, *P. tricornutum* and *T. suecica* extracts consist of 39.34 ± 0.60, 22.94 ± 0.88 and 28.03 ± 1.17 mg/g of polyphenols, respectively. Other species also worth mentioning are *Desmodesmus* sp., *N. salina*, *D. salina* and *N. limnetica,* which can generate lower polyphenol concentrations ranging from 4.52 to 7.72 mg/g. In addition, the above species can provide up to 1.41–4.07 μg/g of ferulic acid [39]. Goiris et al. [34] identified *Arthrospira* sp., *Diacronema* sp., *Phaeodactylum* sp., *Porphyridium* sp., *Tetraselmis* sp., *Chlorella* sp. and *Haematococcus* sp. as producers of ferulic acid in the following concentrations: 0.97 ± 0.12, 2.01 ± 0.25, 0.81 ± 0.009, 0.63 ± 0.007, 0.94 ± 0.11, 0.63 ± 0.007 and 77 0 ± 10 ng/g dry biomass, respectively. This demonstrates that microalgae have the potential to be a source of polyphenols including ferulic acid, and may be applicable in the management of stress and the improvement of mental health.

Microalgae produce a wide range of protective molecules including polyphenols when exposed to a variety of environmental stresses. For example, nutrient control, exposure to low concentration of heavy metals and pH fluctuations all seem to increase microalgal productivity of phenolic acids [96]. Genetic engineering of foods is on the rise, and it has many advantages including increased supply at a lower cost. Recently, eukaryotic gene insertion was carried out in fresh water cyanobacteria *Synechocystis* [97]. The production of ferulic acid by plants, such as grains and berries, is attributed to the presence of a particular set of genes. Plants are eukaryotic organisms which suggest that expression of such sets of genes in cyanobacteria may be achievable, thereby opening up opportunities to increase the production of ferulic acid. 

Ferulic acid demonstrates anti-depressive and anti-inflammatory effects and has impact on stress in vivo. However, its benefits in mental health remain otherwise underexplored. While nutrient manipulation is a strategy to enhance polyphenol content, genome modification in microalgae is unexplored to date for ferulic acid production. It will be of benefit to expand on the current state of knowledge of microalgal polyphenols in stress and mood regulation in clinical trials. 

### 3.5. Quercetin

Flavonoids such as quercetin and anthocyanin malvidin are known to exist in plants including tea, fruit juice, grapes and wine [98,99]. Studies show that flavonoids can exert a neurological response. A quercetin (0.2 mg/kg) and malvidin (5 μg/kg) mixture was administered daily to sleep deprived mice for six weeks. An improvement in cognitive function was observed which was attributed to mediatory effects of flavonoids on signalling pathways, without any undesirable side effects [41]. 

Neuroinflammation prevents correct neuron function and is associated with depression and other neurological disorders [100]. Tayab, Islam and Chowdhury [40] surmised that polyphenols and flavonoids have neuroprotective and anti-neuroinflammation capacity in pre-clinical models and as such have the capacity to modify mood and anxiety and promote positive mental health. In subjects experiencing anxiety, inability to feel pleasure and low mood, symptoms were improved with daily oral administration of 30–50 mg/kg of quercetin for 2–4 weeks. Low locomotor activity, low energy and fatigue are associated with depressed mood and other psychiatric disorders [101]. Subjects supplemented with 25 mg/kg of quercetin per day for four weeks showed increased levels of physical activity [40]. The effects of quercetin on sleep and mental health closely resemble the action of other flavonoids. 

Quercetin, among other flavonoids, is produced by several cyanobacteria and microalgae. Species notably rich in quercetin include *Nostoc ellipsosporum*, *Limnothrix obliqueacuminata*, *Westiellopsis prolifica*, *Microchaete tenera*, which can provide 23.8 ± 1.03, 12.4 ± 0.43, 3.7 ± 0.5 and 18.4 ± 0.85 μg/g fresh wt., respectively. Cyanobacteria *Hapalosiphon fontinalis*, *Calothrix brevissima* and *Phormidium tenue* generate approx. 11 μg/g fresh wt. [35]. Meanwhile, distribution in *Scenedesmus quadricauda* (Chlorophyta), *Diacronema lutheri* (Haptophyta), red microalga *Porphyridium purpureum* and green microalga *Haematococcus lacustris* ranges from 2 to 9.1 ng/g [34,35]. *A. platensis* ethanolic extract is markedly high in flavonoids and can provide 540 μg/g of quercetin [42].

There is a broad range of flavonols distributed in plants, with the highest concentrations found in fruits, red wine and tea. Modifications of microorganisms have been utilised to increase the production of bioactive compounds and other molecules in recent decades, and this approach was applied as previously discussed in the case of quercetin. Naringenin is a precursor to many biomolecules including anthocyanins, flavonols and flavonoids including quercetin. Recently, quercetin was produced by a genetically modified *Saccharomyces cerevisiae* and *Saccharomyces albus* when fed with naringenin [102,103]. The evidence suggests that microorganisms can produce flavonoids and, given the genetic similarities between cyanobacteria and prokaryotes, it is plausible that modified microalgae may also have the capacity to produce quercetin. 

Microalgal quercetin and its role in mental health have not been fully explored to date; however, pre-clinical trials suggest that flavonoids have neuroprotective and anti-neuroinflammatory potential. As previously stated, neuroinflammation is associated with depression and other mental disorders, indicating that the anti-inflammatory action of quercetin could be advantageous. Further studies should consider the effects of quercetin on mental health in clinical trials. 

### 3.6. Hesperidin

Citrus fruits are a source of the bioflavonoid hesperidin which displays antioxidant, anti-inflammatory, sedative and anti-convulsant properties and can improve mood through modulation of neuroinflammation and brain plasticity [43,104,105]. In addition, hesperidin has been shown to be effective in reducing inflammation caused by stress. Oral administration of doses within the range of 20–200 mg/kg for 1–4 weeks to chronically stressed mice significantly elevated depressed mood, reduced inflammation and anxiety, and improved memory and learning capacity [40,43,44]. Hesperidin demonstrates protective outcome against neurodegenerative disorders such as Alzheimer’s disease. Naturally occurring compounds have been successfully used in the development of novel therapeutics in many diseases. Hesperidin appears to be therapeutically involved in signalling pathways for Alzheimer’s and has a potential to serve as a structural template for development of novel therapeutics [106]. 

A broad range of secondary metabolites are produced by several microalgae; however, there is a scarcity of data on the distribution of hesperidin. Green microalgae *C. vulgaris*, *C. hypnosporum* and cyanobacteria *A. platensis* can generate 54.14 ± 0.03, 128.13 ± 0.02 and 51.13 ± 0.0 mg/100 g, respectively [45,46]. In addition, hesperidin was detected in the species of *Nostoc* sp., *Anabaena* sp., *Tolypothrix* sp. and *Chlamydomonas* sp. [45].

Several studies concluded that hesperidin is beneficial for mood regulation, anxiety, cognitive health and neurodegenerative diseases. Flavonoids can be extracted from microalgae, therefore presenting the capacity to serve as a mining source of hesperidin. However, as no data are available on the effects of microalgal flavonoids on mental health, further studies will be beneficial.

### 3.7. Fucosterol

Phytosterols and other sterols are commonly found in cell membrane of plants and cannot be synthesised by humans; therefore, they can only be obtained through diet. Sterols are long established as important for cholesterol regulation and prevention of heart disease. Phytosterol structure is similar to that of cholesterol and can prevent its absorption, hence promoting cardiovascular protection. Studies suggest that phytosterols, in addition to contributing to cardiovascular health, can also contribute to hepatoprotection and immunomodulation [107]. 

Algae are the most abundant marine source of phytosterols, whereas brown algae *Cystoseira foeniculacea* and to some extent red seaweeds are rich in fucosterol [108,109]. Algal fucosterol demonstrates various health benefits such as cell longevity, as well as antioxidative, anti-inflammatory and immunomodulatory action [47,50]. Interestingly, anticholinergic activity of fucosterol causes inhibition of neural plaques associated with Alzheimer’s disease [51]. Evidence also exists that fucosterol can stimulate production of neurotransmitters, hence improving cognitive function [52]. Fucosterol extract isolated from brown algae *Sargassum fusiforme* has been shown to increase neurotransmitters such as serotonin and norepinephrine, resulting in alleviation of low mood in mice (dose 10–40 mg/kg). In addition, fucosterol (20–30 mg/kg) showed an ability to evoke superior anti-depressive response to fluoxetine in vivo [48]. Marine brown seaweed *Ecklonia cava* subsp. *stolonifera* extract containing fucosterol improves cognitive function and inhibits cholinesterase enzymes involved in progression of neurodegenerative disorders [53]. The evidence suggests that fucosterol isolated from marine algae has the potential to act as an anti-depressant and neuromodulator and may offer neuroprotection in degenerative nerve disease. 

To date, few marine microalgae species are known to contain fucosterol; however, some species can accumulate ample phytosterols concentrations. For example, *I. galbana*, *P. tricornutum*, *D. lutheri*, *Tetraselmis* sp. M8 and *Nannochloropsis* sp. BR2 can generate 0.4–3.4% of fucosterol on a dry weight basis. Meanwhile, fucosterol constitutes 1.3% of total sterols found in marine photosynthetic microalgae *O. luteus* and is also distributed in golden-brown microalgae *Chrysoderma* sp., *Chrysomeris* sp., *Chrysowaernella* sp. and *Giraudyopsis* sp. [49]. 

Brown seaweeds and a few microalgae species contain a wide range of phytosterols. Algal phytosterols demonstrate neuro-modulatory and neuroprotective action and the anti-depressive efficacy of fucosterol was reported to be higher than that of the pharmaceutical therapeutic when administered in vivo. Nonetheless, the exact mechanism of phytosterols in mental health remains unclear and requires further detailed in vivo examination.

### 3.8. Carotenoids

Carotenoids are a wide class of secondary metabolites found in various plants, bacteria and some microalgae and fungi [110]. These naturally occurring pigments are associated with numerous health benefits including neuroprotection, mood regulation and improved sleep.

Carotenoids including alpha-carotene, beta-carotene, beta-cryptoxanthin, lycopene and lutein with zeaxanthin were found to decrease depression symptoms and improve low mood in humans. Moreover, higher carotenoid consumption was positively correlated with psychological health [111]. Astaxanthin extracts obtained from the bacterium *Paracoccus carotinifaciens* were administered to adults at a dose of 8 mg/day for 8 weeks. Overall improvement of cognitive ability and memory was reported [112]. Fucoxanthin is another carotenoid which improved sleep patterns among other health effects in animals. Moreover, carotenoids displayed neuroprotective action and neurogenesis in the elderly by regulation of genes responsible for longevity [113]. A mixture of lutein and zeaxanthin (20 mg:4 mg ratio) administered for a 6-month supplementation was shown to increase sleep quality and sleep pattern in a randomised placebo-controlled clinical trial [114]. Moreover, the application of carotenoids decreased the need for pharmacological treatment for insomnia. This suggests that carotenoids may be used as a complementary treatment for sleep regulation. Notably, carotenoid extract from *D. salina* has been shown to exert a neuroprotective effect in vitro and may be beneficial in neurodegenerative disorders such as Alzheimer’s disease [115]. 

Several microalgae species can generate ample concentrations of carotenoids. For example, *H. pluvialis* was found to produce up to 5% of carotenoids per dry weight. Moreover, lutein accounts for up to 80% of total carotenoids in such microalgae [116]. *Nannochloropsis* sp., *P. tricornutum* and *T. suecica* generate up to 5.63 ± 0.11, 5.14 ± 0.05 and 5.62 ± 0.12 mg/g (dry weight), respectively [38]. Several species were also found to provide high concentrations of carotenoids namely *Desmodesmus* sp. (6.70 ± 0.01 mg/g), *D. salina* (4.83 ± 0.01 mg/g), *N. limnetica* (2.56 ± 0.02 mg/g), *N. salina* (5.296 ± 0.01 mg/g) [39]. Carotenoids are beneficial for sleep regulation, neuroprotection and cognitive function. Microalgae are a rich source of carotenoids and can be utilised to produce compounds valuable in brain health.

Carotenoids are fat-soluble molecules and their absorption in the body occurs in a manner comparable to lipids. The permeability of the BBB is crucial to elicit cerebral effect. Several carotenoids exist naturally in the human brain and cell membranes. Carotenoids such as astaxanthin, lutein and zeaxanthin can readily cross the blood–brain barrier [117,118].

The bioavailability of dietary carotenoids is affected by several factors including dietary fibre, some minerals and alcohol. Food constituents may hinder bioavailability of carotenoids by blocking their absorption into the bloodstream through the intestinal wall. Moreover, certain therapeutics including inhibitors of gastrointestinal lipases can inhibit absorption of carotenoids [111]. 

Polar carotenoids can cross cell membranes more readily than non-polar molecules [117]. However, the bioavailability of carotenoids may be enhanced by modification of hydrophobicity of food constituents. The lipophilic nature of carotenoids suggests that their bioavailability would be enhanced when incorporated into lipid rich matrices. Various food formulations, as well as encapsulation, hydrogel implementation and dispersion in an emulsion can be utilised to maximise carotenoid absorption [119,120]. Evidence shows that carotenoids generated by bacteria are more bioavailable and can withstand the harsh environment of the stomach in comparison to other sources [110]. Given the fact that microalgae share certain aspects of genetic make-up with bacteria, it is worth investigating their carotenoid bioavailability vis-à-vis bacteria.

### 3.9. Omega-3 and Other Polyunsaturated Fatty Acids (PUFAs)

PUFAs are long chain fatty acids found in a range of marine organisms such as fish and microalgae, and to some extent in bovine milk [55,121]. Fatty acids and lipids are known to be crucial in correct functioning of tissues, cell membrane structure, receptor function and synaptic plasticity. PUFAs are beneficial for cardiovascular and kidney health, arthritis and development of CNS disorders in children. Certain PUFAs such as α-linoleic (omega-3) and linoleic (omega-6) are not produced by human tissues and need to be obtained through diet [54]. Moreover, PUFAs are associated with decreased neuron inflammation and apoptosis. There is also evidence that omega-3 PUFAs play a critical role in psychiatric health, pathophysiology of neurodegenerative disorders such as Alzheimer’s disease and the protection of the neuron membrane [55,57].

Omega-3 and omega-6 PUFA deficit is associated with sleep difficulties in children [122]. It is established that the quantity ratio of fatty acids in diet is significant for the correct functioning of the brain. For example, omega-3 and omega-6 extract (1:4) was found to restore cognitive function and learning when administered for a duration of four weeks in vivo [123]. On the contrary, another study concluded that higher proportion of omega-3 to omega-6 fatty acids promoted longer sleep time in vivo [124]. Sleep difficulties are frequently reported in patients suffering with Alzheimer’s disease [123]. Administration of an α-linoleic and linoleic fatty acid mixture (1:4) improved sleep cycle regulation in 74% of subjects [54]. Meanwhile, PUFAs were supplemented to sleep deprived rats at concentrations of 2, 4 and 8 μL/g body weight. As a result, an improved cognitive ability and reduced signs of depression were documented [125]. 

Omega-3 PUFA deficit, poor sleep and stress are associated with depression [126]. Omega-3 fatty acid supplementation (1000 mg) for 12 weeks has shown multiple effects in a randomised, double-blind and placebo-controlled study. Increase in positive feelings and ability to cope, improved mood, sleep and anxiety were attributed to the nutraceutical effects of long chain fatty acids [127]. On the contrary, Ross, Malik and Babay [128] concluded that omega-3 PUFA supplementation did not invoke a mood alternating effect in rats fed with omega-3 enriched diet for 8 weeks. Similarly, an 8-week randomised clinical trial concluded that omega-3 fatty acid fish oil supplementation (2000 mg) had no effect on low mood and anxiety symptoms [58]. It was postulated that traditional sources of omega-3 PUFAs cannot close deficiency gaps and promote mental wellbeing [129]. Meanwhile, in another study, omega-3 fatty acid fish oil (100 mg/100 g body weight) was administered to chronically insomniac rats for 8 weeks and brain-protective action was observed [56]. In addition, omega-3 PUFA supplementation at a dose of 1.5 g/day significantly reduced depression symptoms in the elderly [130].

Bioactive compounds formulated as extracts, supplements or as part of a food matrix undergo numerous complex processes once ingested. To allow access of beneficial com-pounds to the brain, digestion into basic molecules is necessary for assimilation into the bloodstream and to allow for transport to target sites. 

Fatty acids are typically metabolised for the most part in the liver before delivery to the brain where they can elicit a desirable effect [131]. For example, compounds produced during the metabolism of marine omega-3 PUFAs from marine fish, such as resolvins D (RvD) and E (RvE), maresins and protectins, provide anti-depressive action by lowering inflammation. These compounds, also called specialised pro-resolving mediators (SPMs), can modulate pro-inflammatory molecules such as cytokines. The immune system produces cytokines as a response to inflammation, infection or injury and they are also important in correct brain function. However, prolonged exposure to cytokines is associated with neuropsychiatric disorders including depression [132]. Moreover, RvE was shown to interact with receptors involved in pro-inflammatory pathways in vivo. Maresins can decrease neuron death and the presence of molecules associated with inflammation. Overall, the biochemical pathways of omega-3 PUFA metabolites were identified as potential therapeutics in neurodegenerative diseases and psychiatric disorders [133]. 

Given that the biochemical pathways of fish PUFAs are well understood, it is antici-pated that fatty acids derived from microalgae will have a comparable mechanism of ac-tion. However, the exact neuroprotective actions of microalgal fatty acids and their role in sleep and depression still remain unresolved and merit further investigation. 

As previously mentioned, there is a strong correlation between inflammation, in-somnia and depression. Inflammatory biomarkers such as IL-6, C-reactive protein, TNF-α and interleukin-2 receptor (sIL-2R) may possibly serve as markers for sleep disturbances and depression. However, identification of psychological disorders through detection of potential biomarkers is still a relatively new area [134,135]. In addition, screening for genetic pre-disposition and gene mutations can possibly be used in prevention of depression, insomnia and anxiety [136].

Microalgae are an excellent source of PUFAs across a wide range of species. Usually, microalgal fatty acids consist of 12–24 carbon in length with a high ratio of PUFAs to monosaturated fatty acids. Microalgae are often overlooked as a source of long chain fatty acids for nutraceutical applications. Nonetheless, species such as *Dunaliella* sp., *T. viridis*, *Nephroselmis* sp., *Cryptomonas* sp. and *Rhodomonas* sp. can produce more than 50% of their total fatty acid content as PUFAs [15]. Overall, omega-3 PUFA content in most microalgae species is higher than 20% of total fatty acids. Microalgal lipid production can be enhanced through environmental manipulation. For example, exploiting light intensity, CO_2_ concentration and temperature can result in the production of up to 85% of total lipids in *Nannochloropsis* sp. [137]. Diatom *P. tricornutum* is able to generate up to 32.2% of its total fatty acids as omega-3. In recent years, intensified lipid production was attempted through genetic modification of microalgae including *P. tricornutum*. As a result of genetic make-up changes, this diatom was able to generate up to 57.8% of total lipids as PUFAs [138]. *Chlorella* (Chlorophyta) and *Schizochytrium limacinum* (Chromista, Labyrinthulomycota) are also abundant in lipids and fatty acids. It was found that *Chlorella vulgaris* can generate up to 40% more lipids when cultivated in a nitrogen-starved environment. Interestingly, green microalgae *Monoraphidium capricornutum* produced higher concentrations of omega-3 PUFAs when cultivated at optimal temperature of 15 °C [139]. 

Notably, the production of microalgal PUFAs is considered more sustainable when compared with aquaculture. Current methods of fish production cannot meet the growing demand for omega-3 fatty acids [140]. PUFAs are crucial for maintenance of healthy sleep patterns, mood stability and brain health. However, there are contradicting data on the effectiveness of omega-3 PUFA supplementation on mood regulation, anxiety and sleep. Nonetheless, microalgae abundant in PUFAs could potentially be employed in the mental health area, maximising this potential by manipulation of growth parameters and genetic enhancement to ensure peak yields. 

In Section 4 below, each of the bioactives identified in Section 3 above will be discussed in terms of their bioavailability and delivery to the target site.

## 4. Bioavailability and Delivery of Microalgae Extracts to the Target Sites

There is little data available on the bioavailability of various microalgae-derived compounds. However, given the similar nature of plant and microalgal phytochemicals such as secondary metabolites, it is probable that the behaviour is comparable. Despite being advantageous for health, several challenges are associated with phytochemical absorption. Similarly, pharmaceutical applications in brain health are not without obstacles. These include drugs for brain cancer, neurodegenerative diseases, stroke and epilepsy [141]. Many therapeutics struggle with the restrictive structure of the BBB and attempts are currently being made to functionalise them as nanostructures to overcome poor permeability. In addition, further impediments such as the presence of plasma protein and enzymes, brain–blood efflux system and the flow of brain blood supply are also an issue in drug delivery [142]. 

Access to the brain structure of microalgae-derived compounds is likely through similar pathways to plant metabolites and pharmaceuticals, and will face analogous obstacles. In recent decades, interest in the optimisation of pharmaceutical and nutraceutical delivery to target sites has picked up in pace. For example, several challenges such as the hydrophobic nature of phytochemical structure and their large size were addressed. Additionally, plant secondary metabolites such as flavonoids, polyphenols and phytosterols have been functionalised with nanocarriers such as micelle vehicles and cyclodextrins as well as solid lipid nanoparticles among others. The following section will focus on bioavailability, dietary nutrient interaction, and overcoming the challenges to delivery to the brain of bioactive compounds found in microalgae. 

### 4.1. β-Phenylethylamine (PEA)

Limited data are available on the bioavailability of PEA in the body and its interactions with dietary compounds. However, it is known that PEA can readily penetrate the BBB and enter the brain from the bloodstream [143]. Studies have shown that PEA can accumulate in the brain and can increase neurotransmitter concentrations such as dopamine, serotonin and norepinephrine and subsequently eliciting an anti-depressive effect. It should be noted that PEA, as well as the above neurotransmitters, are typically broken down by monoamine oxidase B enzyme (MAOB). Inhibition of MAOB results in increased PEA, which is considered an effective form of treatment in Parkinson’s and Alzheimer’s disease. In general, low levels of PEA are seen in depressed individuals and considered to be common in many neurodegenerative disorders [61,144].

Development of water stable PEA/cyclodextrin complex, with the inclusion of gold nanoparticles, was achieved as a potential carrier for pharmaceuticals. High PEA loading is possible with such structures and can ensure efficient delivery of potent concentrations of PEA to the brain. It may be possible to combine psychoactive therapeutics with PEA/cyclodextrin carrier for an enhanced effect [145]. Thus, improved delivery of microalgal PEA could possibly be utilised in mood regulation, sleep and overall mental health. 

### 4.2. Flavonoids

Flavonoids are known for their low solubility and limited bioavailability. Additionally, their large size and polar nature make tissue permeability difficult. Up to 10% of flavonoids are absorbed in the small intestine and further absorption occurs in the large intestine. Gut microbiota play an important role in the metabolism of flavonoids and help in bloodstream assimilation. In the food matrix, flavonoid bioavailability is hindered by the presence of macro and micronutrients. Absorption of flavonoids is limited by proteins, fibre, and minerals, whereas the presence of lipids, fat soluble vitamins and carotenoids and flavonoids increase bioavailability. The hindered bio-accessibility of flavonoids when ingested with foods may be due to the existence of flavonoids enmeshed in the network with other compounds and the restricted access of enzymes to release flavonoids from the food matrix. However, formulation of flavonoids into micelle structures can increase absorption rates [146,147].

Quercetin is known to have low solubility and poor oral bioavailability. Bioavailability of quercetin was increased in vivo upon the addition of certain micronutrients such as pectin, water soluble soyabean fibre and to some extent guar gum [148]. Higher dietary fat content in the food matrix results in higher quercetin absorption. In addition, it was noted that mixtures of lecithin and soyabean oil emulsifiers dramatically increase quercetin solubility [149]. Clinical trials found that novel lecithin-based quercetin (Quercetin Phytosome) increases quercetin plasma concentration up to 20 times. This indicates a promising outcome for flavonoid bioavailability with lecithin-based quercetin formulations [150].

Flavonoids can exert a synergistic effect on one another in vitro. Quercetin bioavailability can be increased up to three times by addition of apigenin. Moreover, apigenin absorption can be enhanced by 50% in the presence of quercetin. In addition, vitamin E (α-tocopherol) was found to enhance plasma and brain concentration of flavonoids including quercetin in vivo. Phenolic compounds, namely catechins and flavonoids such as naringenin, decrease quercetin intestinal permeability, hence decreasing plasma concertation in vivo [146]. On the other hand, lipids mostly pass through the cell membrane with little difficulty. It is expected that coating flavonoids with a lipid layer may be the answer to overcoming low solubility and absorption. Formulation of phytonutrients as nanostructures or nanoemulsions allow delivery to target sites which native large structures cannot reach. 

#### Strategies for Enhanced Delivery of Flavonoids

Nanostructures often include inorganic particles such as Superparamagnetic Iron Oxide and Gold/Silver systems, and organic nanocarriers such as micelle vehicles and solid lipid nanoparticles. The encapsulation ensures stability, permeability and delivery of the bioactive to the desired site with minimum loss of its bioactive capacity [146,147]. Moreover, structural modifications such as methylation and glycosylation of flavonoids can alter their intestinal bioavailability [151]. Increased solubility and permeability of hesperidin in the small intestine can also be achieved [152]. 

Hesperidin incorporated in a gold nanostructure was effective as a drug treatment in cancer therapy in mice [153]. Furthermore, cyclodextrins are oligosaccharides which can exert a protective effect on molecules. Formulation of cyclodextrin/flavonoid complex improves solubility of insoluble flavonoids and increases their bioavailability. Similarly, enhanced delivery was reported using co-crystal systems which are often used to increase oral bioavailability of pharmaceuticals. Co-crystalisation of flavonoids with coformers such as caffeine, proline and betaine, among others, was attempted in recent years with promising results. Molecules that enhance absorption such as fatty acids, bile salts, emulsifiers, chitosan and chelating agents are often employed to increase efficacy of pharmaceuticals. It is possible that similar methods may be used for delivery of poorly water-soluble microalgal flavonoids, thus maximising the effect on brain health [154]. 

### 4.3. Polyphenols

Polyphenolic compounds demonstrate low solubility in the intestinal tract and accordingly present with low bioavailability which limits their nutraceutical applications. Other challenges associated with the application of polyphenols are their large structures and difficulty with access to the target site. It was found that dietary polyphenols can cross the BBB to some extent and accumulate in the brain. However, polyphenols are prone to efflux pump and are easily eliminated and in addition have poor absorption rates and low solubility at the BBB [155,156].

Adam et al. [157] showed that food matrix interactions, predominantly with polysaccharide networks in cereals, limit the bioavailability of supplemented ferulic acid in rats. In fact, complex food matrices hinder bioavailability of polyphenols in general [157]. However, bioavailability of ferulic acid from less complex products such as beer is as high as 98%. It appears that ferulic acid bioavailability is associated with the complexity of the food matrix and its bio-accessibility [158]. 

#### Functionalisation of Polyphenols for Improved Absorption

Technologies tasked with functionalisation of food constituents often involve transformation processes to ensure maximised bioavailability. Such alterations of physio-chemical properties of phytochemicals, with the use of enzymes or applications of compound delivery vehicles, have been achieved. The use of feruloyl estrases to release ferulic acid from the food matrices has the potential to contribute towards increased bioavailability [159]. For example, polyphenolic compounds such as resveratrol have been shown to have improved bioavailability in the brain when delivered in a nanoparticle-assisted manner [160]. Functionalisation of antioxidants and other bioactives, such as keratin nanofibers carriers, increases compound stability and solubility. Bioavailability of polyphenols and catechins from tea was increased upon delivery using liposomal colloidal carriers. Similarly, encapsulation of resveratrol with nanoemulsions and casein-loaded nanoparticles showed superior bioavailability and stability [156]. Microalgal polyphenols may potentially be distributed to the brain in an analogous approach, which would enhance its capacity to exert desirable effects in the brain. 

### 4.4. Fucosterol

The bioavailability of phytosterols is governed by their crystalline structure, degree of saturation, chain length, source, delivery route as well as ingested food matrix [89]. Marine algae are abundant in phytosterols, whereas fucosterol is produced by several microalgae. Fucosterol isolated from the brown macroalga *Sargassum fusiforme* demonstrates extremely low absorption of up to 0.74% [89]. Phytosterols in general are known to have slow and poor solubility and absorption rates (up to 5%); however, they can accumulate in tissues in small concentrations [89]. Phytosterols were found to penetrate the BBB, concentrate in the brain and may therefore elicit neuroprotective action in neurodegenerative diseases [161]. Evidence suggests that, because of their poor bioavailability, dietary phytosterols cannot reach sufficient concentration in tissues to promote positive health outcomes. 

#### Novel Approaches for Phytosterol Delivery

To overcome limitations of phytosterols, such as low absorption, several strategies were proposed. For example, droplet size reduction, which can be achieved through nanoencapsulation, was recommended as one of the mechanisms to enhance the timely release, oral bioavailability and stability of phytosterols. Nanostructured lipid carriers for colloidal delivery and cyclodextrin inclusion complexes may also be employed to increase solubility and bioavailability of phytosterols. In addition, phytosterols coupled with catechin or quercetin exert complementary action on one another, resulting in increased bioactivity and efficacy [162].

### 4.5. Omega-3 PUFAs

Although omega-3 fatty acids were identified as desirable compounds for sleep and mental health, their limited efficacy remains an ongoing issue. There has been an interest in recent decades in the improvement of omega-3 PUFA bioavailability. For instance, it was proposed that combinations of omega-3 PUFAs with polyphenols may be beneficial for increased effect on the brain. This is unsurprising given that the interactions of dietary compounds within the food matrix have been long established. Moreover, fatty acids are metabolised in the small intestine often in the presence of bile salts which exert a surfactant role on lipid molecules [163].

Although the BBB fulfils a crucial cerebral protection role, this poses an additional hurdle for pharmaceuticals as well as nutraceuticals such as PUFAs. In addition, the BBB is responsible for elongation and desaturation of omega-3 and omega-6 fatty acids. This process promotes the creation of longer fatty chains which are desirable for the correct functioning of the brain. There are frequent interactions among fatty acids and other molecules at the BBB site. For example, cholesterol is known to cause neuronal membrane hardening, which decreases BBB permeability and was found to reduce uptake of omega-3 PUFAs. In contrast, omega-3 and omega-6 fatty acids are advantageous for membrane fluidity and BBB integrity. PUFAs appear to exert a cooperative relationship on the BBB which directly benefits the brain [54].

#### Transformations of Omega-3 PUFAs for Increased Bioavailability

To elicit an effect, omega-3 PUFAs must penetrate the BBB. It can be safely assumed that fatty acids being hydrophobic in nature and of relatively small size may cross these membranes. Indeed, there is evidence that fatty acids, including omega-3 fatty acids, can pass through the BBB and reach the brain. Usually, the efficacy of fatty acids absorption can reach up to 90% [164]. However, dietary omega-3 PUFAs often exist in a triglyceride or ethyl ester form which may hinder the absorption. One of the proposed solutions to overcome poor bloodstream assimilation is to orally distribute esterified omega-3 in a monoglyceride form [165]. Moreover, incorporation of omega-3 fatty acids in an emulsion of microdroplets can maximize absorption. Advanced Lipid Technologies (ALT) and Self-Micro Emulsifying Delivery System (SMEDS) are novel methods of formulating fats into micelles for enhanced bioavailability and delivery to target sites [166].

Given the high absorption rates of fatty acids, it is likely that microalgal omega-3 fatty acids can potentially accumulate in the brain. While PUFA bioavailability can be increased through novel approaches, further research is required to elucidate the neuro-modulatory effects of microalgal omega-3 PUFAs. 

### 4.6. Fermentation as a Biological Functionalization of Microalgal Biomass

Enhancing the functionality of microalgae secondary metabolites may extend beyond the physical synthesis of nanomaterials. The use of lactic acid bacteria (LAB) in fermentation has been used extensively as a method of food preservation and of late to enhance the potential applications of nutrients such as bioactive peptides for health benefits [167]. Biological functionalisation of whole microalgal biomass through fermentation has been attempted. Implementation of probiotic *Lactobacillus plantarum* to ferment dried *A. platensis* led to interesting developments in terms of bioactive properties of microalgae. It was reported that the antioxidative activity and concentrations of phenolic compounds were notably improved by up to 79% and 320%, respectively [168]. These reported observations can be attributed to LAB breaking down the polyphenolic structure of the biomass and further releasing subunits with both an enhanced bioactivity and a reduced overall size. It is worth investigating the levels of BBB penetration that these sub molecules might have. *L. plantarum* also demonstrated the capacity to release bioactive peptides, fatty acids and other secondary metabolites of *A. platensis* [169].

Biological functionalization such as fermentation could be employed to produce microalgal derivatives implicated in sleep and mental health such as flavonoids, polyphenols, fatty acids and phytosterols. The previously compiled evidence suggests that these bioactives are often too large to access the target sites in concentrations sufficient for a desirable effect. Using targeted lactic acid and microalgal species for enhanced production of functional metabolites could provide a new opportunity for nutraceutical applications in the treatment of sleep mental disorders. These metabolites could be delivered in the form of pharmaceutical base supplements.

**Figure 1:**
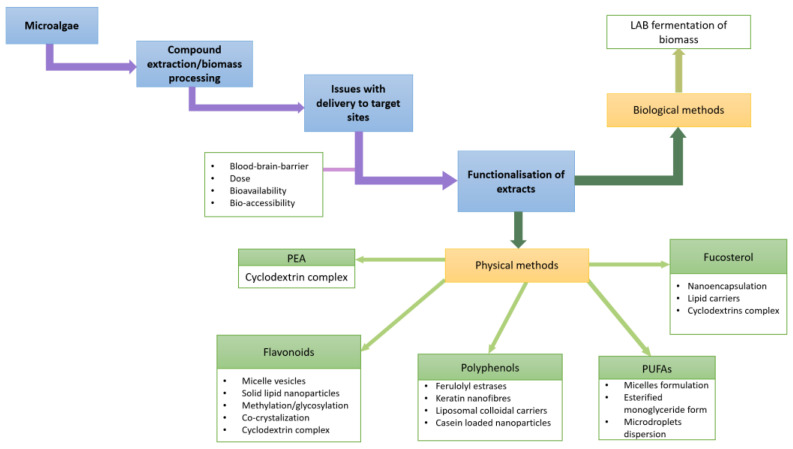
Schematic of functionalization of microalgae bioactives as discussed in Section 4.

Figure 1 is a schematic illustrating the main concepts described in Section 4.

## 5. Methodology

The search strategy focused on the latest publications in relation to the effect of microalgae on sleep disorders, depression and related stress and anxiety disorders. Databases consulted include Science Direct, Scopus, Web of Sciences and the Directory of Open Access Journals. Over 200 peer reviewed publications were consulted. The search was carried out using the following keywords: “microalgae”, “bioactive compounds”, “polyunsaturated fatty acids”, “sleep and sleep disorders”, “mood regulation” and “stress”. Articles noting clinical trials connecting microalgae biomass and bioactives to sleep and neuropsychiatric disorders were also included in the search. Given the nature of the present review and the current limited level of research addressing the impact of microalgae bioactives on CNS disorders, the search was focused on incorporating as much related information as possible.

## 6. Conclusions and Future Perspectives

Microalgae are an abundant sustainable source of various secondary metabolites. Microalgal biomass and its derived compounds such as PEA, flavonoids, polyphenols, phytosterols and long chain fatty acids have been shown to be capable of inducing sedation, sleep and mood regulation as well as neuroprotection and neuro-modulatory action. 

Challenges associated with the application of microalgal extracts in the area of brain health are mainly associated with limited bioavailability of bioactives. The ability of microalgal derivatives to cross the intestinal wall and the blood–brain–barrier is key. For instance, due to their size, polyphenols have difficulties with membrane permeability and are also prone to efflux pump. Although the evidence shows that microalgae extracts can achieve therapeutic effects in a dose-dependent manner, hindered bioavailability has a direct effect on the dose that can reach the target site. Bioactivity of microalgae and its derivatives can be affected by conditions applied during harvesting and extraction processes. Such procedures may affect structural stability and cause the compounds to lose their health-promoting properties. An improved understanding of the issues associated with bioavailability of microalgae-derived compounds is therefore worth exploring.

Interactions between dietary compounds within the food matrix appear to be an important factor in the therapeutic effect of microalgal compounds. In some cases, compounds can exhibit a synergistic effect, leading to enhanced bio-accessibility, as is the case with polyphenols, which enhances the absorption of omega-3 PUFAs. 

Several approaches are currently being examined to increase the potential of secondary metabolites from plants, bacteria and microalgae in health applications. As one example, polyphenols have the issue that their large structure prohibits their prompt delivery into the brain. In addition, phytosterols are characterized with low solubility and absorption, and cannot accumulate in the brain tissue at levels sufficient to elicit desirable response. In order to counteract these issues, encapsulation, incorporation of emulsions, micelles and hydrogels are among many approaches directed towards drug delivery to targeted sites in the brain, thereby enabling enhanced bioavailability of the bioactive compounds. The above technologies have advanced significantly in recent times, thus presenting real opportunities for the investigation of microalgae bioactives in brain health. 

Detailed mechanisms of action of microalgae-derived compounds in sleep, mood regulation, neuroprotection and neuro-modulatory action are not yet available. There is currently a lack of human studies into the psychoactive effects of microalgae. However, the outcome of in vitro studies to date is promising. The establishment of high-quality clinical trials will contribute invaluably to the critical understanding of specific processes and therapeutic potential of microalgae in sleep and mental health. 

Microalgal ingredients may potentially be applied as complementary therapies, thus reducing the adverse side-effects of the pharmaceuticals typically administered today. Microalgae offer an interesting possibility as cell bio-factories, making them attractive for compound mining. There is a strong opportunity for a multi-disciplinary approach to explore the properties of microalgae bioactives in brain health applications and this review aims to initiate research and development in this area. 

## Figures and Tables

**Table 1:** Microalgae and their compounds in sleep, mood regulation, anxiety and stress.

Effect	Microalgal Biomass/Bioactive Compound	Microalgae	Reference
Neuroprotection	Whole biomass	*Arthrospira platensis*	[29]
Prevention of cellular oxidative damage			
Decreased inflammation			
Neuroprotection	Whole biomass	*Chlorella*	[30]
Prevention of cellular oxidative damage			
Inflammation lowering activity			
Oxygen generation in CNS	Cultures	*Chlamydomonas reinhardtii*	[31]
Shorter sleep latency	Whole biomass	*Euglena gracilis*	[32]
Mood improvement			
Ability to wind down			
Calming of the sympathetic nervous system			
Improvement in social interaction, focus and concentration			
Sleep cycle regulation	β-Phenylethylamine	*Aphanizomenon flos-aquae* (Klamin^®^ extract)	[33]
Anti-depressive effect			
Neuron regeneration			
Antioxidative and anti-inflammatory action			
Norepinephrine elevating action	Apigenin	*Arthrospira platensis*	[34,35]
		*Chlorella vulgaris*	
Anti-depressive effect		*Diacronema lutheri*	
		*Haematococcus lacustris*	
Sleep cycle regulation		*Leptolyngbya* sp.	
		*Phaeodactylum tricornutum*	
Sedative effect		*Porphyridium purpureum*	
		*Tetraselmis suecica*	
Mood regulation	Ferulic acid	*Arthrospira* sp.,	[34,36,37,38,39,40]
		*Chlorella* sp.	
Anti-depressive action		*Desmodesmus* sp.	
		*Diacronema* sp.	
Stress lowering activity		*Dunaliella salina*	
		*Haematococcus* sp.	
Anti-inflammatory action		*Nannochloropsis* sp.	
		*Phaeodactylum* sp.	
Increased norepinephrine concentration		*Porphyridium* sp.	
		*Tetraselmis* sp.	
Improvement in cognitive function, locomotor activity and mood regulation	Quercetin	*Arthrospira platensis*	[34,35,40,41,42]
		*Calothrix brevissima*	
		*Diacronema* *lutheri*	
		*Haematococcus pluvialis*	
		*Hapalosiphon fontinalis*	
		*Limnothrix obliqueacuminata*	
		*Microchaete tenera*	
		*Nostoc ellipsosporum*	
		*Phormidium tenue*	
		*Porphyridium* *purpureum*	
		*Scenedesmus quadricauda*	
		*Westiellopsis prolifica*	
Anti-depressive action	Hesperidin	*Arthrospira platensis*	[40,43,44,45,46]
		*Anabaena* sp.	
Reduced inflammation and anxiety		*Chlorella vulgaris*	
		*Chlamydomonas* sp.	
Improvement memory and learning ability		*Chlorococcum hypnosporum*	
		*Tolypothrix* sp.	
Anti-depressive, neuro-modulatory and neuroprotective action	Fucosterol	*Chrysoderma* sp.	[47,48,49,50,51,52,53]
		*Chrysomeris* sp.	
Anti-inflammatory and anticholinergic action		*Chrysowaernella* sp.	
		*Ecklonia cava* subsp. *stolonifera* extract	
Elevated serotonin and norepinephrine concentrations		*Isochrysis galbana*	
		*Nannochloropsis* sp. BR2	
Improvement of cognitive function		*Olisthodiscus luteus*	
		*Diacronema lutheri*	
Promotion of cell longevity		*Phaeodactylum* *tricornutum*	
		*Tetraselmis* sp. M8	
Correct functioning of membranes	PUFAs including omega-3 fatty acids	*Cryptomonas* sp.	[15,54,55,56,57,58]
Improvement in synaptic activity		*Dunaliella* sp.	
Neuroprotective action		*Nephroselmis* sp.	
Decreased neuroinflammation		*Rhodomonas* sp.	
Sleep promoting action		*Tetraselmis viridis*	
Improvement of cognitive ability			
Mood regulation			
Anti-depressive action			
Anxiety lowering effects			
